# A role for the stringent response in ciprofloxacin resistance in *Pseudomonas aeruginosa*

**DOI:** 10.1038/s41598-024-59188-z

**Published:** 2024-04-13

**Authors:** Libertad García-Villada, Natalya P. Degtyareva, Ashley M. Brooks, Joanna B. Goldberg, Paul W. Doetsch

**Affiliations:** 1 Genomic Integrity and Structural Biology Laboratory, NIEHS, Durham, NC USA; 2 Integrative Bioinformatics, Biostatistics and Computational Biology Branch, NIEHS, Durham, NC USA; 3 Department of Pediatrics, Emory University School of Medicine, Atlanta, GA USA

**Keywords:** Antimicrobial resistance, Bacterial genetics

## Abstract

*Pseudomonas aeruginosa* is a major cause of nosocomial infections and the leading cause of chronic lung infections in cystic fibrosis and chronic obstructive pulmonary disease patients. Antibiotic treatment remains challenging because *P. aeruginosa* is resistant to high concentrations of antibiotics and has a remarkable ability to acquire mutations conferring resistance to multiple groups of antimicrobial agents. Here we report that when *P. aeruginosa* is plated on ciprofloxacin (cipro) plates, the majority of cipro-resistant (ciproR) colonies observed at and after 48 h of incubation carry mutations in genes related to the Stringent Response (SR). Mutations in one of the major SR components, *spoT,* were present in approximately 40% of the ciproR isolates. Compared to the wild-type strain, most of these isolates had decreased growth rate, longer lag phase and altered intracellular ppGpp content. Also, 75% of all sequenced mutations were insertions and deletions, with short deletions being the most frequently occurring mutation type. We present evidence that most of the observed mutations are induced on the selective plates in a subpopulation of cells that are not instantly killed by cipro. Our results suggests that the SR may be an important contributor to antibiotic resistance acquisition in *P. aeruginosa*.

## Introduction

*Pseudomonas aeruginosa* is a Gram-negative bacterium usually found in locations associated with human activity^1^. It is a major cause of nosocomial infections, including burn infections, urinary tract infection, and pneumonia^2^. It is also the leading cause of chronic lung infections in cystic fibrosis and chronic obstructive pulmonary disease patients^3,4^. Antibiotic treatment remains challenging because *P. aeruginosa* is resistant to high concentrations of antibiotics and has a remarkable ability to acquire mutations conferring resistance to antimicrobial agents^5–8^. Such intrinsic resistance is multifactorial, involving physical, physiological, and genetic determinants specific to *P. aeruginosa*, whereas resistance acquired through mutations is driven by the repeated exposure of the bacteria to high concentrations of antibiotics^9^.

During a chronic infection, bacteria are exposed not only to antibiotics but also to reactive oxygen species (ROS). These ROS are generated within the bacterial cells by their own metabolism^10,11^. They are also produced by the immune system in response to infection^12,13^. An increasing number of studies suggest that oxidative DNA damage, which can induce mutations, plays an important role in antibiotic resistance development^10^ and progression^14^.

The mutagenic effect of oxidizing agents on *P. aeruginosa* has not been studied in depth. Also, published works on this topic have been carried out with growing cultures, while most bacteria during infection are usually in a slow- or non-growth state^15,16^. We were interested in studying the mutagenic effect of these agents in stationary phase *P. aeruginosa* populations. To avoid the effect of phenotypic lag, we grew our experimental cultures in minimal medium at 30 °C, since under these conditions most *P. aeruginosa* cells are expected to have only one copy of the chromosome^17^. The experimental cultures were exposed to oxidizing agents that bacteria encounter while interacting with the immune system: nitric oxide (NO), hypochlorous acid (HClO), and hydrogen peroxide (H_2_O_2_)^12,18^. To select for mutants, we used ciprofloxacin (cipro), a fluoroquinolone commonly prescribed for the treatment of chronic *P. aeruginosa* infections^19^. It functions by inhibiting DNA re-ligation by DNA gyrase and topoisomerase IV during DNA unwinding, thus producing double-strand DNA breaks that impede DNA replication and cell division, and that, if not repaired, cause cell death^20^.

As shown in several studies, resistance to cipro in *P. aeruginosa* is often caused by base substitutions (BSs) in *gyrA* and *nfxB*^21–24^, which encode, respectively, the GyrA subunit of the DNA gyrase, and NfxB, a repressor of the multidrug efflux operon *mexCD*–*oprJ*. However, our results indicate that most *P. aeruginosa* cipro resistant (ciproR) mutants owe their resistance to mutations in genes related to the Stringent Response (SR), a bacterial stress response in reaction to amino-acid starvation, fatty acid limitation, iron limitation, heat shock and other challenging conditions. These results imply that mutations that affect the SR may be an important source of antibiotic resistance in *P. aeruginosa*.

## Results

### Most ciproR mutants carry mutations in genes related to the SR

Exposure of wild-type (WT) *P. aeruginosa* strain MPAO1 to HClO at 1 mg/ml for 60 min led to a ~ 10–19-fold increase in mutants resistant to 2 μg/ml of cipro (Supplementary Fig. S1). Surprisingly, none of the sequenced ciproR mutants (Sanger sequencing: 38 from untreated and 75 from HClO-treated cultures), isolated at 48 h of incubation, carried a mutation in *gyrA*. In addition, ciproR colonies were small and grew slowly, in contrast to the typical *gyrA* ciproR colonies, which are larger and readily detectable after less than or at 48 h of incubation^22^. To map the genomic location of the mutations, whole genome sequencing (WGS) was performed on a sample of the resistant colonies (20 from untreated and 40 from HClO-treated cultures).

Table 1 lists the genes with mutations and their corresponding incidences. These mutations were detected in > 90% of the reads. Some isolates had additional DNA changes detected in 65% or less reads. The description of these other mutations (which likely occurred during the propagation of the resistant clones in the presence of cipro) is included in the Supplementary File S1.

**Table 1:** Mutated genes in ciproR isolates from untreated and HClO-treated cultures.

Mutated gene(s)^a^	Untreated	HClO-treated
*spoT*	11 (48%)	14 (34%)
Aminoacyl-tRNA-synthetase(s)	5 (22%)	13 (32%)
*rpoN*	1 (4%)	4 (10%)
*gyrA/B*	1 (4%)	3 (7%)
Other	3 (22%)	7 (17%)
Total^b^	21	41

^a^For each gene or category, the values shown are the total number of mutations detected and the respective relative percentage (in parenthesis).

^b^20 and 40 ciproR mutants were sequenced from untreated and HClO-treated cultures, respectively.

About 70% of the mutations were located in genes related to the SR. The most frequent were mutations in *spoT*, comprising about 40% of all mutations. *spoT* codes for SpoT, a guanosine-3',5'-bispyrophosphate (ppGpp) 3'-pyrophosphohydrolase known to be the primary alarmone of the SR. It synthesizes ppGpp during carbon, phosphate, iron, and fatty acid starvation, and it also hydrolyzes ppGpp, thus providing an essential function for the regulation of ppGpp levels in the cell^25,26^. *P. aeruginosa spoT* knockout mutants have an increased intracellular ppGpp content^27^, a change in metabolism that in *Escherichia coli* is known to be associated with activation of the SR^28^. Of the 26 mutations we observed in *spoT*, 16 are insertions and deletions (InDels) that change the reading frame, and one is a nonsense mutation (Supplementary Files S1 and S2). Since these mutations likely lead to partly- or non-functional SpoT, we hypothesized that they also induce the activation of the SR through ppGpp accumulation.

Mutations were also frequently found in various aminoacyl-tRNA synthetase (aaRS) genes. These were: *tRNA-Val*, *argS*, *glyS*, *alaS*, *tsaB*, *pheT*, *ileS*, *serS*, *valS*, *ilvN*, and *glyQ*. Most of these genes are considered essential for *P. aeruginosa*^29–31^. Accordingly, all except two of the mutations we observed in these genes are BSs and short deletions that do not change the reading frame (Supplementary File S1) and may therefore not completely eliminate the functions of their products. Spontaneous and targeted mutations in different aaRS genes activate the SR in *E. coli*^32,33^. In addition, the L-serine analog L-serine hydroxamate, which inhibits the enzyme seryl-tRNA synthetase, is used in vitro to induce increased intracellular ppGpp content in bacteria, including *P. aeruginosa*^34^, through amino acid starvation.

Mutations were also detected in *rpoN*, which codes for the transcription factor sigma 54. Some reports suggest that, in *P. aeruginosa*, *rpoN* contributes to the SR^35–37^. However, *rpoN* deletion mutants do not have this stress response activated^38^. The mutations we observed in this gene were frameshifting (Supplementary File S1) and therefore probably knockouts.

Mutations were also found in the genes that code for the DNA gyrase subunits *gyrA* and *gyrB*. One mutation was observed in *gyrA* (Supplementary File S1). In combination with other mutations, changes in *gyrB* were observed in eight different isolates. As expected, all mutations found in these essential genes are nonsynonymous BSs or in-frame InDels.

Other mutated genes were (number of occurrences in parenthesis): *tuf* (2), *rpoB* (1), *siaA* (1), and *ssg* (1). Also, one isolate contained a large duplication (18,219 bp long), and four isolates had long deletions (from 153,140 to 439,822 bp long) that involved different genes (Supplementary File S1). Deletions such as these, that extend up to over 620 kb, have been reported for *P. aeruginosa* and shown to confer phage resistance^39,40^.

### Mutations conferring cipro resistance in *P. aeruginosa* are most likely induced by cipro

The most frequent mutations in the ciproR mutants that we sequenced were InDels, in particular deletions that encompass more than 1 base pair (Table 2). They ranged from 2 to 33 base pairs. This result was unexpected since the spectrum of spontaneous mutations for *P. aeruginosa* mostly comprises BSs^41^. In addition, HClO induces mainly C to T transitions^42^.

**Table 2:** Mutation spectrum of ciproR isolates from untreated and HClO-treated cultures.

Mutation class^a^	Untreated	HClO-treated
Base pair substitutions	2 (10%)	9 (22%)
Insertions/Deletions	17 (80%)	30 (73%)
+ /− 1 bp	1	7
Insertions > 1 bp	4	3
Deletions > 1 bp	12	20
Other	2 (10%)	2 (5%)
No mutations detected	1	4

^a^For each type of mutation, the values shown are the total number of mutations detected. Relative percentages are also shown (in parenthesis) for Base pair substitutions, Insertions/Deletions, and Other.

Cipro is known to induce mutagenesis in bacteria^43^. Experiments in *Staphylococcus aureus*^44^ and *E. coli*^43,45^ with sublethal and lethal exposures to this antibacterial show that it causes a high proportion of small InDels, mostly comprised in deletions. Since a significant fraction of the ciproR mutants we sequenced carried InDels, we wondered if they have not been merely selected by cipro, but rather, induced and selected by it, and decided to test this hypothesis. Thus, we repeated the selection experiments (without exposing the cultures to any oxidizing agent) and monitored the appearance of ciproR colonies on the selective plates for a few days after the first 48 h of incubation. Then we analyzed the distribution of resistant colonies among the plates.

We reasoned that if the mutants were generated upon exposure to cipro, they should originate from a subpopulation of cells that do not die in the presence of 2 μg/ml of the antibiotic, or at least not immediately, and in that case, it may be expected that resistant colonies would continue to appear over time, as reported for *E. coli*^43^. To verify that the mutants were of the same nature as those we observed in previous experiments, we sequenced *spoT*, since the WGS showed that about 40% of the mutations present in ciproR mutants were in this gene.

**Figure 1:**
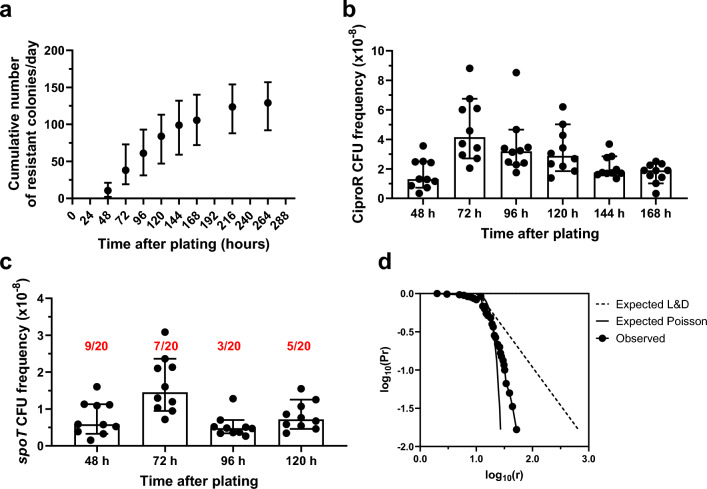
Evidence for induction of ciproR mutants by cipro. *P. aeruginosa* WT strain MPAO1 cultures were grown in minimal medium and then plated on selective plates containing 2 μg/ml cipro. (**a**) Cumulative number of new ciproR colonies on the selective plates over 11 days after plating. Counting of new colonies were performed every 24 h or 48 h after the first 48 h of incubation. Symbols represent median and range of n = 10 selective plates. (**b**) Frequency of new ciproR colonies per day on the selective plates for days 2 to 7 after plating. Frequencies were estimated as new colonies per day/total CFU plated. Bars and error bars represent median and 95% CI of n = 10 selective plates. (**c**) Frequency of new *spoT*, ciproR colonies per day on the selective plates for days 2 to 5 after plating. Frequencies were determined by multiplying the observed proportion of new *spoT* mutants per day by the frequencies in (**b**). Bars and error bars represent median and 95% CI of n = 10 selective plates. Numbers in red represent the proportion of ciproR isolates with a *spoT* mutation detected at each of the indicated times after plating. (**d**) Expected and observed distribution of ciproR colonies on the selective plates. Dash-dotted line represents expected Luria and Delbrück (L&D) distribution calculated with the assumption that resistant mutants originate during bacterial growth before plating. Solid line depicts expected Poisson distribution calculated with the assumption that resistant mutants originate on the selective plates. The observed distribution (each dot represents an experimental measurement) is based on a total of 1215 colonies scored over 6 days on 10 selective plates. Pr represents the probability that a culture has r or more mutants. Results for 1 of 3 independent experiments (other results are available in the supplemental material). See text for additional details.

Figure 1 reports the results of a representative selection experiment (see additional results in Supplementary Fig. S2). As expected, resistant colonies accumulated on the selective plates for at least 11 days after plating (Fig. 1a). Yet, their frequency decreased gradually (Fig. 1b), which may indicate that the subpopulation from which the mutants originate dies over time. Every day for the first five days after plating, we isolated a random sample of new colonies from the selective plates and sequenced *spoT*. The proportion of *spoT* mutants in the samples varied from day to day (Fig. 1c), likely due to the small size of the samples, but their presence and types of mutations (predominantly InDels) suggested that the resistant colonies appearing over time on the selective plates were of the same nature as those observed in previous selection experiments at 48 h of incubation.

For the analysis of the contribution of cipro exposure to mutagenesis on the plates, the distribution of colonies among the selective plates over seven days of incubation was represented in a log10(Pr) versus log10(r) graph (where P is the proportion of cultures that contain r or more mutants; see Materials and Methods for details) and compared to the distributions expected if the mutants pre-existed before being plated (expected Luria and Delbrück: due to the stochasticity of spontaneous mutagenesis, the frequency of mutants varies significantly among parallel, independent cultures) or originated on the selective plates (expected Poisson: mutagenesis takes place according to a specific rate and thus the frequency of mutants is similar among parallel, independent cultures). The distribution of colonies among the selective plates was very close to the expected distribution if the resistant mutants would have originated on the selective plates (Poisson distribution) (Fig. 1d). Because of this result and the high proportion of InDels, we concluded that the antibacterial present on the medium likely contribute to mutation accumulation.

The results reported so far come from experiments performed with the WT strain MPAO1. This strain is the parental strain of the widely utilized transposon insertion mutant library from the University of Washington^46^. It is also a descendant of the strain PAO1, which is used as model in most labs that experiment with *P. aeruginosa*. Despite their relatedness, there are significant genetic differences between these strains^47–49^. Thus, we checked that our results were not exclusive to MPAO1. Supplemental Figure S3 show results of representative selection experiments performed with PAO1. As with MPAO1, ciproR colonies appeared on the selective plates over several days after plating and most of them seemed to originate on the plates.

### Evidence of selection of persisters by cipro

When a *P. aeruginosa* population is exposed to a concentration of cipro several times its MIC, a subpopulation of cells, tolerant to the lethal effect of the antibacterial, will survive^50^. These tolerant cells are called persisters. Persisters are a subpopulation of nongrowing, non-replicative, dormant bacteria that exhibit transient high levels of tolerance to antibiotics^51^. They are the source of resistant cells that can restore a bacterial population after being exposed to an antibiotic at a lethal concentration^52,53^. In our experiments, we used a cipro concentration of 2 μg/ml, which, under our experimental conditions, is 2 × MIC (Supplementary Table S1). To assess the possibility of presence of persisters at this concentration, first we determined the minimal bactericidal concentration (MBC) for the WT strains (MPAO1 and PAO1). Results showed that, under our experimental conditions, 2 μg/ml of cipro kills most cells but leaves alive a variable percentage of survivors (Supplementary Fig. S4). This kind of fluctuations in numbers of surviving cells occur when rich medium is used to grow the experimental cultures^52^, and may explain the variability in ciproR mutant frequency in different selection experiments.

**Figure 2:**
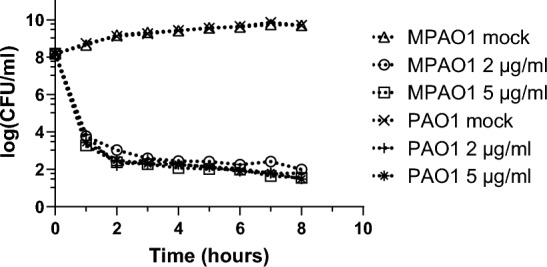
Time-kill curve in the presence of 2 μg/ml cipro is biphasic. MPAO1 and PAO1 cultures were exposed to 2 and 5 μg/ml cipro and their growth kinetics were followed for 8 h. Both cipro concentrations render the same biphasic time-kill curve. Symbols represent the median of n = 3. Errors bars (95% CI) are smaller than the corresponding symbols and thus no visible.

Next, we determined if a biphasic killing pattern, which is characteristic of antibiotic persistence^54^, can be seen when sensitive cultures are exposed to 2 μg/ml cipro. Figure 2 represents the data obtained from three independent time-kill assays conducted with MPAO1 and PAO1. It shows a biphasic killing curve with a steep slope reflecting fast killing followed by a plateau, which suggests that not all the bacteria in the population are killed at the same rate. Indeed, most of the cells are killed during the first hour of incubation, and the remaining die very slowly over the following hours. The biphasic killing curve obtained at 2 μg/ml cipro was not different from the curve obtained at 5 μg/ml cipro, which is 5 × MIC for cipro for our WT strains.

Finally, we asked if the survivors in the MBC and the time-kill experiments when cultures were exposed to 2 μg/ml cipro should be classified as persisters. To this end, we isolated a random sample of survivors and determined their MIC and cell survival following an additional exposure to 2 μg/ml of cipro. If in the first round of selection the ciproR cells were mutants (not persisters), they all will be resistant and continue growing in the presence of cipro. If these survivors were persisters (not mutants), the expectation would be that, when exposed de novo to a lethal concentration of cipro, the proportion of survivors will be similar to the percentage of survivors in the initial experiment, when they were exposed to such concentration for the first time. Accordingly, their MIC also should not change. Our results (Supplementary Fig. S5) suggest that both types of cells (persisters and real mutants) are selected at 2 μg/ml cipro. Yet, these results are difficult to interpret since the observed mutants may be the result of cipro-induced mutagenesis.

### Standard selection delays but does not prevent the appearance of SR mutants

In selection experiments comparable to ours, most ciproR colonies isolated at 48 h of incubation will owe their resistance to BSs in *gyrA* or *nfxB*^22,23^. Similar experiments with *E. coli* indicate that such mutations are generated spontaneously during the growth of the cultures, before plating on the selective plates^43^. We were puzzled by why our results were so dissimilar to these reports. The main differences between our experimental protocol and the protocols of other studies are the conditions in which the cells are grown before being plated, and the number of cells plated. Typically, for selection experiments, cultures are grown in rich medium and, thus, they reach a cell density significantly higher (about ten-fold) than when they are grown in minimal medium, which is the medium that we used. This is probably why we did not observe *gyrA* ciproR mutants. The frequency of these mutants is very low (of the order of 10^–9^;^22,23^) and we plated only about 7 × 10^8^ cells per plate. The odds of observing *gyrA* mutants was thus low. Even when cells are grown in rich medium, most of the cultures will be devoid of such mutants.

To determine if selection of mutations in SR-related genes is favored by our experimental conditions, we repeated the selection experiments, this time following a standard protocol. Thus, cultures were grown in LB for 24 h at 37 °C before being plated on cipro plates (in previous experiments, they were grown in M9 medium for 48 h at 30 °C). At 48 h of incubation on the selective plates, we observed large, thick resistant colonies that grew fast. From experience, we suspected that most of these large colonies likely carried mutations in *gyrA*. Yet, the following day, after 72 h of incubation, the small, slow-growing colonies become visible on the plates and were the only type that continue appearing on a daily basis until the end of the experiments.

**Figure 3:**
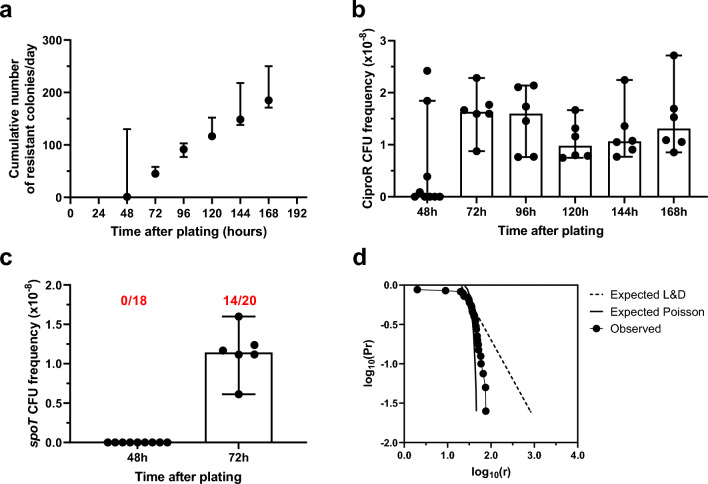
Standard selection delays but does not prevent the appearance of SR mutants. *P. aeruginosa* WT strain MPAO1 cultures were grown in LB and then plated on selective plates containing 2 μg/ml cipro. (**a**) Cumulative number of new ciproR colonies on the selective plates over 7 days after plating. Counting of new colonies were performed every 24 h after the first 48 h of incubation. Symbols represent median and range of n = 10 or 6 selective plates. (**b**) Frequency of new ciproR colonies per day on the selective plates for days 2 to 7 after plating. Frequencies were estimated as new colonies per day/total CFU. Bars and error bars represent median and 95% CI of n = 10 or 6 selective plates. (**c**) Frequency of new *spoT*, ciproR colonies per day on the selective plates for days 2 and 3 after plating. Frequencies were estimated by multiplying the proportion of new *spoT* mutants per day by the frequencies in (**b**). Bars and error bars represent median and 95% CI of n = 10 and 6 selective plates. Numbers in red represent the proportion of ciproR isolates with a *spoT* mutation detected at each of the indicated times after plating. (**d**) Expected and observed distribution of ciproR colonies on the selective plates. Dash-dotted line represents expected Luria and Delbrück (L&D) distribution calculated with the assumption that resistant mutants originate during bacterial growth before plating. Solid line depicts expected Poisson distribution calculated with the assumption that resistant mutants originate on the selective plates. The observed distribution (each dot represents an experimental measurement) is based on a total of 1427 colonies scored over 6 days in a total of 10 or 6 selective plates. Pr represents the probability that a culture has r or more mutants. Results for 1 of 2 independent experiments (other results are available in the supplemental material). See text for additional details.

Results of one representative experiment are shown in Fig. 3 (additional results are included in the Supplementary Fig. S6). As observed previously with cells grown in M9 medium (Fig. 1), resistant colonies accumulated on the cipro plates for at least 11 days (Fig. 3a). Yet, in these selection experiments, there was not an obvious decrease in their frequency over time (Fig. 3b), which is probably an additional effect of using a different protocol.

CiproR colonies isolated at 48 h of incubation did not carry mutations in *spoT* (Fig. 3c). We sequenced *gyrA* of the corresponding isolates. Seventeen out of 18 colonies isolated at 48 h of incubation carried a mutation in this region, and all these mutations were single BSs. So, the results were similar to those reported by other groups for this type of selection experiment. However, *spoT* mutants started to appear on the third day after plating, indicating that ciproR mutants generated after cipro exposure also appear on the selective plates when following a standard protocol (Fig. 3d), albeit requiring a longer time for them to develop.

The same results were obtained for the strain PAO1 (Supplementary Fig. S7).

### CiproR mutants have impaired growth and altered intracellular ppGpp content

**Figure 4:**
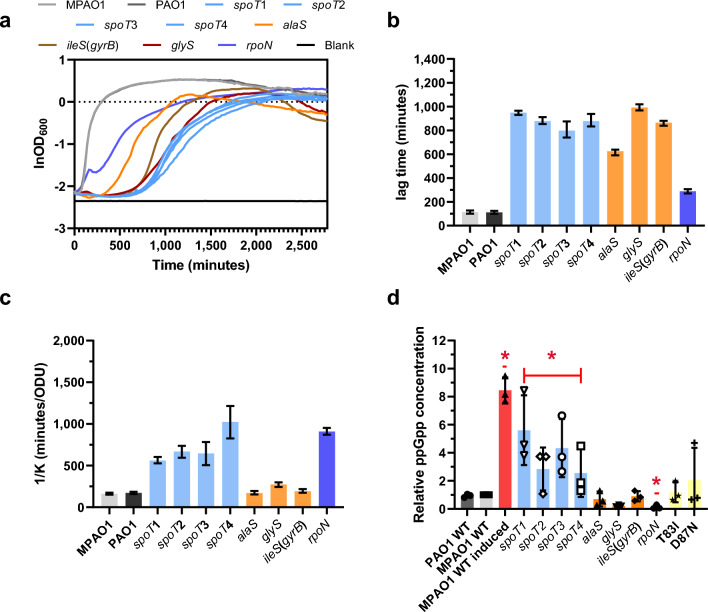
CiproR mutants have impaired growth and altered intracellular ppGpp content. (**a**) Growth curves of WT and different ciproR mutants. Lag time (**b**) and growth rate (**c**) derived from the growth curves in panel A. (**d**) Intracellular ppGpp concentration is shown relative to the WT strain MPAO1. WT cells were exposed to serine hydroxamate (SHX, 1 mg/ml final concentration) as a positive control for ppGpp accumulation (MPAO1 WT induced). *gyrA* mutants T83I and D87N were used as controls of cipro exposure (see Materials and Methods for additional details). In (**a**), data shown are the means of n = 8 technical replicates; in (**b**) and (**c**), bars and error bars represent the means and 95% CI of n = 8 technical replicates; in (**d**), bars and error bars represent the means and SD of n = 3 independent experiments. Red asterisks indicate statistically significant differences between means, as determined by t-test (different types of mutants and induced WT compared to non-induced WT; PAO1 and MPAO1 combined, all *spoT* combined, all aaRS combined, *gyrA* mutants combined; *P* < 0.05).

Due to its effect on the bacterial metabolism (it decreases the levels of cellular transcription, specifically of genes involved in the biosynthesis of macromolecules), activation of the SR impacts bacterial growth, reducing it to a minimum^55^. To confirm SR activation in the newly discovered ciproR mutants, we first determined the length of the lag period and the growth rate of several sequenced ciproR mutants. We chose four *spoT* mutants with mutations in different locations of the gene (*spoT*1, *spoT*2, *spoT*3, *spoT*4); three aaRS mutants (*glyS*, *ileS*, *alaS*); and one *rpoN* mutant (Supplementary File S1). Figure 4b, c show, respectively, lag time and growth rate (expressed as the reverse of the curve rate constant) determined from one representative growth curve (Fig. 4a). Overall, these results were in agreement with the observed properties of the ciproR colonies we isolated on the selective plates.

We also determined the intracellular mid-log ppGpp content of the same sample of mutants. High intracellular ppGpp levels are a sign of SR activation. Figure 4d shows that the intracellular ppGpp content was altered in most of the ciproR mutants. All *spoT* mutants showed increased averaged content when compared with the WT strain (MPAO1). They also showed high variability in ppGpp content among independent experiments. This variability may be due, in these slow-growing cultures, to the accumulation of suppressor mutants that decrease the ppGpp concentration. None of the tested aaRS mutants showed increased ppGpp content. The *rpoN* mutant showed a significantly lower amount of ppGpp. In summary, ppGpp appears to accumulate in the *spoT* mutants but not in the other ciproR mutants tested.

To confirm that the observed mutations in SR-related genes confer resistance to cipro, we determined the MIC for cipro of the same set of mutants described above and conducted complementation experiments (Supplementary Table S1 and Fig. S8). To test whether the observed resistance was specific to cipro, we also determined the MIC of other clinically relevant antibiotics: rifampicin (inhibits transcription), tetracycline (inhibits translation), gentamicin (inhibits translation), and imipenem (inhibits cell wall synthesis). Our results (Supplementary Table S1) indicated that the ciproR mutants tested are four to eight times more resistant to cipro than the WT strains (MPAO1 and PAO1). Yet, none of them showed increased resistance to any of the other antibiotics used. These data suggest that the mechanism of resistance of the observed ciproR mutants is specific to cipro (and probably similar fluoroquinolones).

Restoration of cipro sensitivity through complementation with the WT *spoT* and *alaS* genes in the corresponding mutants was partial for some isolates and total for others (Supplementary Table S1). Partial complementation may be due to inhibition of the WT protein activity by the mutated version or to a lower level of WT gene expression, compared to the mutant allele. It may also be due to the appearance, during the construction of the complemented strains, of secondary mutations in other genes that also confer cipro resistance (since ciproR mutants were always grown in LB with 2 μg/ml of cipro to keep the resistance). Nevertheless, growth was completely restored for all the *spoT* mutants whereas the complemented *alaS* strain still showed a slightly increase of the lag time (Supplementary Fig. S8).

### CiproR mutants display a spoT hot spot mutation in a ATGGCC triple repeat

**Figure 5:**
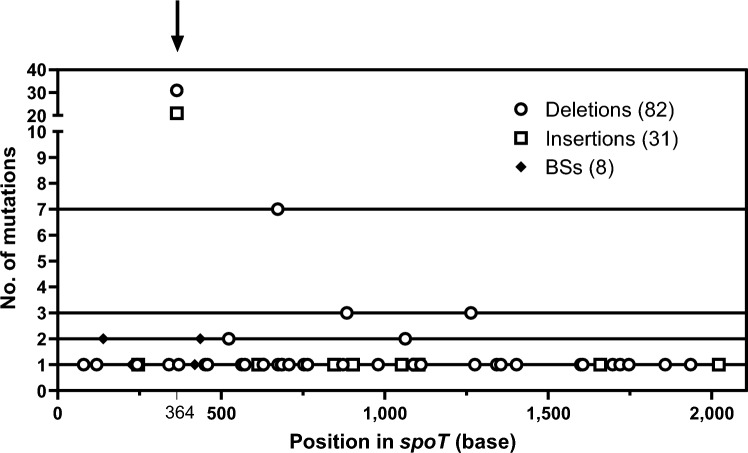
Location of the mutations detected in *spoT* in ciproR isolates. The *spoT* gene of a total of 485 random ciproR isolates was sequenced, 121 turned out to have a mutation in the gene. The black arrow indicates the location of the main hot spot observed in the gene, at positions 384- 381. BSs stands for base substitutions.

During the performance of the selection experiments, we sequenced *spoT* in 485 ciproR isolates. One hundred and twenty-two of them contained a mutation in this gene. Figure 5 shows the spectrum of *spoT* mutations (see additional information in Supplementary File S2). Although we found mutations across the whole length of the gene, there are two hot spots that contained most of them. One at positions 364–381, and another at positions 673–699. The first one is composed of a triple repeat of the sequence ATGGCC. At this hot spot, we scored 30 –ATGGCC, 1 –GCCATG, 19 +ATGGCC, and 2 +GCCATG. For the second hot spot, we scored 11 deletions of different sizes (from 10 to 27 bases). A close look at each mutation reveals that many of them are located in between direct repeats (Supplementary File S2), which indicates the occurrence of slipped misalignment during DNA replication at these locations. This is also a characteristic of cipro-induced mutations^45^.

## Discussion

We show here that, in *P. aeruginosa*, ciproR mutants that accumulate over time on cipro selective plates carry mutations in different genes related to the SR (Table 1). We provide evidence that exposure to cipro on the plates contribute to the generation of these mutations (Figs. 1 and 3, and Supplementary Figs. S2, S3, S6, and S7). SR-related mutants have impaired growth and an altered content of ppGpp per cell when compared to the WT strain (Fig. 4).

Viducic et al.^27^ showed that an engineered partial deletion of *spoT* in *P. aeruginosa* renders a mutant strain with significantly increased amounts of ppGpp per cell compared to the WT strain. Although the MIC for cipro was the same for both strains, the *spoT* mutant strain had a higher tolerance to cipro than the WT strain. Tolerance or resistance to fluoroquinolones can also be acquired through mutations in aaRS genes and other genes involved in DNA translation, as shown by several studies in *E. coli*^32,33,56^. Such traits seem to depend, at least partially, on RelA, the main *E. coli* ppGpp synthetase. To our knowledge, similar reports for *P. aeruginosa* do not exist, but our results indicate that mutations in these genes also confer resistance to cipro in this bacterium. The mechanism of cipro resistance in aaRS mutants requires further investigation since our results show that they do not induce an increased accumulation of ppGpp (Fig. 4d).

Some of the ciproR mutants carried mutations in *rpoN*, which encodes the transcription factor sigma 54. This factor plays an important role in the nitrogen metabolism, motility, mucoidy, and quorum-sensing system. Viducic et al.^38^ demonstrated that *rpoN* knockout mutants of *P. aeruginosa* are more resistant to cipro than their parental, WT strain. This effect is partly dependent on increased *vqsR* expression (*vqsR* codes for VqsR, which controls the expression of the genes required for pyoverdine biosynthesis) and pyoverdine production^38^. Yet, the relationship between *rpoN* and antibiotic resistance is complex and not well understood^57^. The differential growth of the *rpoN* mutants when compared with the *spoT* or *aaRS* mutants (Fig. 4a, b) suggests that mechanisms of cipro resistance may differ. In addition, these mutants showed a significant decrease in the ppGpp content per cell (Fig. 4d), which indicates that their resistance is not due to an activation of the SR.

We have defined, for the first time, a mutation spectrum of ciproR mutations for *spoT*. Mutations were found along the entire gene, but there is a clear hot spot at the position 364–381 (which is located in the hydrolase domain), in the place of a triple direct repeat of ATGGCC (Fig. 5). Mutations in this region are characterized by the loss or gain of an ATGGCC repeat. This suggests the occurrence of slipped misalignment during DNA replication at this locus^58^. There are other locations in the gene in which mutations occurred between direct repeats (Supplementary File S2). These kinds of mutations are characteristic of cipro-induced mutagenesis^45^. The mechanism by which they are generated was investigated by Song et al.^45^ in *E. coli* and, more recently, by Mercolino et al.^59^ in *P. aeruginosa*. Even though it was not possible to elucidate which pathways of DNA metabolism were responsible for them, the Song et al. report indicates that these mutations tend to occur in GC-rich regions, which are prone to acquire secondary structures that may favor the occurrence of slipped misalignment.

From our results, it remains unclear why the spectrum of mutations in untreated cultures did not differ from the HClO-treated cells. One explanation is that HClO-treated cultures showed an increased mutagenesis because the very same subpopulation that survived the lethal effect of the antibacterial was also more resistant to the effect of the oxidant.

Activation of the SR and antibiotic tolerance and resistance can be induced in *P. aeruginosa* and other bacteria though designed mutations in *spoT* or in aaRS^27,32,33,56^. In addition, inactivation of the SR through deletion mutations in *relA* and *spoT* render cells more sensitive to the effect of different antibiotics^60^. The role that the SR may play in the tolerance and resistance to antibiotic treatments of pathogenic bacteria has become a matter of interest and study over the past several years^60–65^. Recent studies indicate that epigenetic changes leading to the tolerance of antibiotics (in contrast to genetic changes) is a step preceding the development of resistance in bacterial infections, and the SR seems to be an important component in this step^60,66–70^. In *P. aeruginosa* at least, the SR seems to confer tolerance by reducing the levels of oxidant stress induced by some antibiotics^68,71^.

Here we show that in *P. aeruginosa* resistance to cipro may take place through mutations in different genes, many of them related to the SR. In most cases, such mutations induce ppGpp accumulation in the cells, which, according to the literature, indicates activation of the SR. However, mutants of the type we report here have been overlooked when mutations in resistance genes have been reported in WGS studies of *P. aerugino*sa clinical isolates^72,73^. Investigators usually pay attention to those mutations that are well known to confer resistance under laboratory conditions. Yet, little is known about how resistance to antibiotics starts during an infection. *gyrA* mutations confer high resistance to cipro with little cell-growth effect, but their frequency is very low, while mutations in SR-related genes, according to our results, seem to be much more frequent. At the beginning of an infection, when the number of infecting cells is low, we think these mutations may play an important role. Even if they are outcompeted or suppressed by other (secondary) mutations afterwards. We also show that ciproR mutants may be generated upon exposure to a lethal concentration of cipro in a subpopulation of tolerant cells. These results argue that targeting the cell effectors that make cells persisters may be another way of combating antibiotic resistance, as suggested by recent reports^51,53,70,74,75^.

## Materials and methods

### Bacterial strains and growth media

The strain MPAO1 was obtained from the *Pseudomonas aeruginosa* Two-Allele Library (Manoil Lab; University of Washington Genome Sciences, Seattle, WA^76^), and the strain PAO1 belongs to the Goldberg Lab’s bacterial collection (Emory University, Atlanta, GA). The PAO1 *gyrA* mutant strains were constructed by transferring the plasmids carrying mutant *gyrA* alleles by two-step allelic exchange^77^. Mutations were introduced to the WT *gyrA* gene cloned into pEx18Tc plasmid by overlap extension PCR cloning^78^.

Cultures were grown in Luria Bertani (LB) medium with a NaCl concentration of 5 g/L, or in M9 medium (M9 minimal salts (Gibco) supplemented with MgSO4 (2 mM), CaCl2 (100 μM), and sodium citrate tribasic (0.25%)). Cells were always plated on LB agar (bacto agar 2%). Selective plates for the selection experiments contained 2 μg/ml of ciprofloxacin hydrochloride (MP Biomedicals).

### Selection experiments (SE)

For the SE, cells were streaked from a frozen stock on LB plates and incubated for 24 h at 37 °C. Next day, a culture was started in LB medium with a single colony and grown at 37 °C with shaking. After 18 h, the culture was diluted 10^5^-fold in M9 medium, and 5-ml aliquots were distributed in a series of 10 culture tubes and incubated at 30 °C with shaking. After 48 h, the experimental cultures were washed once with M9 salts. From each culture, a 2-ml aliquot was taken and spun, and its cells plated on a selective plate and incubated at 37 °C. Resistant colonies were scored at 48 h of incubation and then every 24 h for 7 days. Experimental cultures were also serially diluted, and samples were plated on LB agar to determine the cell density of the cultures. When LB medium was used as growth medium, experimental cultures were diluted 10^5^-fold in LB medium and grown for 24 h at 37 °C with shaking, and only a 1-ml aliquot was plated on the selective plates. In SE in which mutagenesis was induced with hypochlorous acid (HClO; sodium hypochlorite solution, Sigma), experimental cultures were each started with an independent colony and cells were grown in M9 medium; after being washed with M9 salts, two 2-ml aliquots were taken from each experimental culture: one was used as control, the other was exposed to 1 mg/ml HClO. Aliquots were incubated at 37 °C with shaking. After one hour of incubation, aliquots were washed twice and a sample of each was serially diluted in M9 salts and plated on LB agar to determine their cell density; the remaining of each aliquot was spun and their cells plated on independent selective plates. In all experiments, new resistant colonies were picked from the plates at random at the indicated times (48, 72, 96, and 120 h after incubation) and independently placed in culture tubes with 5 ml of LB with 2 μg/ml cipro. These cultures were grown overnight at 37 °C with shaking and next day a 0.6 ml aliquot of each was mixed with 0.4 ml of a 50% glycerol solution in a freezing vial and stored at −80 °C.

### Library preparation and whole-genome sequencing

DNA was extracted using a Maxwell RSC Cultured Cells DNA Kit (Promega) and its quality determined in an Agilent 5200 Fragment Analyzer System using a Genomic DNA 50 kb Kit (Agilent). Indexed pair-end libraries were prepared with TruSeq DNA Library Prep Kit (Illumina Inc, USA) and sequenced with NextSeq 550 System Mid-Output Kit (Illumina Inc, USA) in a NextSeq 500 System Whole-Genome Sequencing Solution (Illumina Inc, USA). The resulting paired-end reads were 150 bp long.

### Variant calling

Two independent analytical pipelines, CLC Genomics Workbench 20 and in-house methods, were employed to generate a high-quality variant set.

With CLC Genomics, reads were first trimmed and then aligned to the MPAO1 reference genome (GenBank CP027857). Once aligned, removal of duplicates and local realignment of reads around InDels were performed with default settings. For variant calling, the minimum central quality was 30, the minimum neighborhood quality was 20, and two minimum variant allele frequency (VAF) threshold were used: 25 and 35. For InDels and structural variants, the P-value threshold was 0.0001 and the minimum number of reads, 2.

To apply the in-house analysis pipeline, the quality of raw sequencing reads was assessed with the FastQC software^79^. Trim-Galore! v.0.4.4 was used to remove Illumina adapter sequences (http://www.bioinformatics.babraham.ac.uk/projects/trim_galore/). Reads with phred score quality < 30 and length < 30 bp after trimming were discarded. The trimmed reads were aligned to the MPAO1 reference genome with the BWA MEM algorithm v.0.7.15 at default parameters^80^. The Samtools v.1.3.1 rmdup function was applied to remove duplicate reads^81^. The alignment rate ranged from 95.9 to 98.2% (median = 96.9%) and the average coverage across all samples was 38X. Data on the reads count and percentage of alignment is summarized in Supplementary File S3. Single nucleotide variants (SNVs) and insertion or deletions (indels) were called with the Bcftools v.1.4 mpileup and Bcftools call functions^82,83^. Raw variants were filtered at a minimum quality score of 100 and at least 10 reads of support for InDels. The Ensembl Variant Effect Predictor (VEP) v.105.0 was used to annotate variants^84^.

All variants were manually checked. In addition, aligned reads were visually checked for the presence of duplications and deletions.

Sequence files have been deposited in the Sequence Read Archive (SRA) under study number PRJNA1010205 and BioSample accession numbers SAMN37183447 to SAMN37183507.

### Expected distributions of mutants

The expected Luria and Delbruck distribution of ciproR colonies was estimated using the formula Pr = 2*m/r, where m is the probable number of mutations per culture that gave rise to the observed distribution of ciproR colonies/day, and r is the observed number of colonies/selective plate/day^85^. m was estimated for all the distribution of colonies as the Lea and Coulson method of the median^86^. The expected Poisson distribution was estimated by multiplying the number of colony scorings (plates x days scored) by the probability mass function of a Poisson distribution. Applied to our data, this function is defined as Pr = λke-λ/k!, where λ is the mean of ciproR colonies/selective plate/day, and k is class of resistant colonies/selective plate/day.

### ppGpp quantification

Cultures were started directly in tryptic soy broth from frozen stocks and grown at 37 °C. CiproR mutants and *gyrA* mutants (used as controls of cipro exposure) were grown in the presence of 2 μg/ml of cipro to keep their cipro resistance. MPAO1 exposed to 1 mg/ml final concentration of L-serine hydroxamate (Molecular Toxicology Inc.) for one hour was used as positive control of ppGpp accumulation. When cultures reached an OD600 ~ 0.6, cells were harvested by centrifugation and stored at -80 °C until analysis.

Solid phase extraction was performed on Oasis WAX (Waters, 1 mL, 10 mg, 30 µm) mixed mode weak anion exchange cartridges. Cartridges were conditioned with 1 mL 0.5% phosphoric acid in 90% acetonitrile, 1 mL 90% acetonitrile, and 1 mL ammonium formate buffer. The 0.4 mL sample aliquots were diluted with 0.6 mL ammonium formate buffer and loaded onto SPE cartridges. Loaded samples were washed with 1 mL ammonium formate buffer and 1 mL methanol. Samples were eluted with 1 mL of 1:2:7 28% ammonium hydroxide:methanol:water mixture into disposable glass centrifuge tubes.

Samples were dried overnight under vacuum in a CentriVap Concentrator (Labconco). Dried samples were reconstituted in 20 µL 0.1% acetic acid in water followed by 80 µL 10 mM triethylammonium acetate in methanol. Samples were transferred to deactivated glass inserts in autosampler vials. An injection volume of 2 µL was used for LC–MS/MS analysis.

Liquid chromatography was performed on an InfinityLab Poroshell 120 HILIC-Z column (Agilent, 2.1 × 150 mm, 2.7 µm, PEEK lined). Mobile phase A was 10 mM ammonium acetate, pH 9, with 2 mL/L InfinityLab Deactivator Additive and mobile phase B was 10 mM ammonium acetate, pH 9, in 90% acetonitrile. A Sciex ExionLC system was used. The LC system and column were flushed with 0.1% phosphoric acid in 90% acetonitrile overnight prior to G4P analysis. Analytical flow rate was 0.4 mL/minute. Mobile phase composition started at 75% B and was adjusted linearly to 40% B at 6 min. The composition was held at 40% B for 2 min then the column was equilibrated with 75% B for 6 min prior to the next analysis. G4P eluted at a retention time of 2.04 min.

Tandem mass spectrometry was performed with a Sciex 6500 + equipped with a Turbo V IonSpray source. Source settings were Curtain Gas 45, CAD Gas High, IonSpray 4000 V, Temperature 550 °C, Gas 1 40, Gas 2 55, Declustering Potential 140 V, and Entrance Potential 10 V. Positive ion electrospray ionization was used. G4P was detected using the [M + H] + ion at m/z 604.0 in Q1 and the m/z 152.1 fragment in Q3 with the collision energy set to 35 V. A confirmation fragment of m/z 502.0 was also monitored with the collision energy set to 25 V. Response was linear over a 7-point calibration ranging from 0.01 to 10 µM (the ppGpp standard used was from Trilink Biotechnologies, Inc.).

### Growth curve analysis

Cultures of ciproR mutants were started directly in LB with 2 μg/ml cipro from frozen stocks and grown at 37 °C for 42 h with shaking. WT strains MPAO1 and PAO1 were grown also from frozen stocks in LB at 37 °C for 18 h; complemented strains were grown the same way but with 100 μg/ml of tetracycline. CiproR mutants and WT and complemented strains were incubated for different periods (42 h vs. 18 h) before the experiments because the mutants grow much slower (as shown in Fig. 4) and require more time to reach saturation. Then, all cultures were diluted in fresh LB medium to an OD_600_ of ~ 0.05 and 8 × 200 μl aliquots for each strain were independently placed in the wells of a 96-well cell culture plate (Costar). 200 μl of fresh LB medium were placed in 16 wells that were used as control wells. Plates were incubated at 37 °C with shaking in a multimode microplate reader (Tecan Spark) and A_600_ measurements were taken every 20 min for 47 h.

For each strain, the resulting A_600_ values were fitted to a curve using a “plateau followed by one phase association” equation in GraphPad Prism 9 (GraphPad Software Inc., U.S.A.), and the lag length and the growth constant, and their corresponding 95% CI, obtained as best fit X0 and Tau values, respectively.

Both, growth curves and genetic complementation experiments, were performed twice, each of them with eight technical isolates per strain.

### MIC and MBC determinations

MIC determinations were performed according to a standard protocol^87^ with a few modifications. In brief, mutant and wild-type cultures were grown as detailed in the previous section and then diluted in fresh LB media to a density of about 5 × 10^6^ cells/ml. 4 μl-aliquots of each culture with about 10^4^ bacteria were spotted onto a series of LB agar plates. For the ciproR mutants, the plates contained the following cipro concentrations: 0, 1.0, 2.0, 3.0, 4.0, 5.0, 6.0, 7.0, 8.0, 10.0, 12.0 14.0, 16.0 and 20.0 μg/ml. Agar plates were then incubated at 37 °C for 48 h before checking the presence of bacterial growth in the spots through visual inspection. For the MIC determinations done for the WT strains, cultures were started from independent colonies and selective plates contained the following cipro concentrations: 0, 0.0625, 0.125, 0.250, 0.5, 1.0, and 2.0 μg/ml. In these experiments, selective plates were incubated for 24 h before checking the presence of bacterial growth in the spots.

The protocol was the same for all the other antibiotics tested, although the concentrations used on the plates varied among the different antibiotics: with tetracycline and rifampicin we tested 5, 10, 15, 20, 25, 30, 35, and 40 μg/ml of the corresponding antibiotic; with gentamicin 0.5, 1.0, 1.5, 2.0, 2.5, 3.0, 3.5, 4.0 μg/ml; and with imipenem 1, 2, 3, 4, 5, 6, 7, 8, 10 μg/ml.

MIC experiments were performed at least three times for each antibiotic.

For the MBC determinations, WT cultures were started from independent colonies in LB medium and grown for 18 h. Then cultures were diluted in fresh LB medium to a density of 10^5^ cells/ml and incubated at 37 °C for 18 h with the following concentrations of cipro (not all the concentrations were used every time this experiment was performed): 0, 0.25, 0.5, 1.0, 2.0, 2.5, 3.0, 4.0, and 8.0 μg/ml. Samples of the cultures were diluted in M9 salts and plated on LB agar at time 0 and at the end of the incubation time to determine the effect of the different concentrations of cipro on cell growth.

### Assessing persister cells

For the time-kill curves, an independent isolate of each WT strain, MPAO1 and PAO1, was grown for 18 h at 37 °C in LB medium with shaking and then diluted 100-fold in fresh medium. Cultures were grown till they reach an OD_600_ ~ 0.4. Then each culture was split into three flasks (mock, 2 μg/ml, and 5 μg/ml cipro) that were incubated at 37 °C with shaking for 8 h. Every hour, 1-ml samples were taken from each flask, washed once with M9 salts and an appropriated dilution was plated on LB agar. Plates were incubated for 24 h at 37 °C before colonies were scored. This experiment was performed three times.

Assessing of persisters cells was conducted as per Hazan et al.^88^. In brief, five independent isolates of each WT strain, MPAO1 and PAO1, were exposed to 2 μg/ml of cipro as detailed in the previous section for the MBC experiments. After 18 h of incubation, 1 ml of each culture was washed with M9 salts and plated on LB agar. Plates were incubated for 24 h at 37 °C before colonies were scored. The percentage of survivors was determined with respect to the total number of cells per culture at the beginning of the experiment (time 0). Two colonies were isolated per experimental culture, grown overnight in LB medium and exposed again to 2 μg/ml as previously to determine their percentage of survivors. In addition, their MIC was also determined, as detailed in the previous section.

### Complementation of *spoT* and *alaS*

A construct composed of *spoT* with its promotor sequence was obtained through gene synthesis (Genewiz) and digested with SacI/HindIII. *alaS* with its promotor sequence was amplified by PCR (the sequences of the primers alaS_PCR-F and alaS_PCR-R are listed in the Supplementary Table S2) and the product was purified and digested with SacI/XbaI. Both digestion products were independently ligated into a pUCP18Ω-Tc^89^ plasmid that had also been digested with SacI/XbaI to obtain plasmids pUCPST and pUCPAS, respectively. These plasmids were transformed into MAX Efficiency DH5 Competent Cells. Five independent transformants were selected on LB agar plates containing 10 μg/ml of tetracycline and their plasmid purified and Sanger sequenced (the sequences of the primers used in this step are listed in the Supplementary Table S2) to verify the absence of mutations. pUCPST was then electroporated into strains *spoT*1, *spoT*2, *spoT*3, and *spoT*4, and pCCPAS was electroporated into strain *alaS*. Transformants were selected on LB agar plates containing 100 μg/ml of tetracycline. Complementation of *spoT* and *alaS* deficiency was demonstrated by the reversion of the increased cipro-resistance and impaired growth of the corresponding mutants. Experiments were performed three times, each with a different isolate of each transformant.

### Sanger sequencing

As indicated before, samples of resistant colonies were isolated from the selective plates of the SE and grown overnight in LB + 2 μg/ml cipro to create frozen stocks. A sample from these overnight cultures was used to amplify the *spoT* (in some cases, also gyrA) through PCR, and then the amplification products were Sanger sequenced (GENEWIZ) and analyzed for mutations using CLC Genomics Workbench 20.

The whole-genome sequencing results obtained for the ciproR mutants characterized in this study were confirmed with Sanger sequencing (GENEWIZ).

A list of all the primers used for Sanger sequencing is included in the Supplementary Table S2.

### Supplementary Information

Supplementary Information 1.

Supplementary Information 2.

Supplementary Information 3.

Supplementary Information 4.

## Data Availability

Sequence files that support the findings of this study have been deposited in the Sequence Read Archive (SRA) under study number PRJNA1010205 (https://www.ncbi.nlm.nih.gov/sra/?term=PRJNA1010205) and BioSample accession numbers SAMN37183447 to SAMN37183507 (https://www.ncbi.nlm.nih.gov/biosample?LinkName=bioproject_biosample_all&from_uid=1010205).

## References

[1] Crone S (2020). The environmental occurrence of *Pseudomonas aeruginosa*. APMIS.

[2] Lister PD, Wolter DJ, Hanson ND (2009). Antibacterial-resistant *Pseudomonas aeruginosa*: Clinical impact and complex regulation of chromosomally encoded resistance mechanisms. Clin Microbiol Rev.

[3] Garcia-Clemente M (2020). Impact of *Pseudomonas aeruginosa* infection on patients with chronic inflammatory airway diseases. J Clin Med.

[4] Rossi E (2021). *Pseudomonas aeruginosa* adaptation and evolution in patients with cystic fibrosis. Nat Rev Microbiol.

[5] Morita Y, Tomida J, Kawamura Y (2014). Responses of *Pseudomonas aeruginosa* to antimicrobials. Front Microbiol.

[6] El Zowalaty ME (2015). *Pseudomonas aeruginosa*: Arsenal of resistance mechanisms, decades of changing resistance profiles, and future antimicrobial therapies. Future Microbiol.

[7] Murray JL, Kwon T, Marcotte EM, Whiteley M (2015). Intrinsic antimicrobial resistance determinants in the superbug *Pseudomonas aeruginosa*. mBio.

[8] Botelho J, Grosso F, Peixe L (2019). Antibiotic resistance in *Pseudomonas aeruginosa*: Mechanisms, epidemiology and evolution. Drug Resist Updat.

[9] Ciofu O, Tolker-Nielsen T (2019). Tolerance and resistance of *Pseudomonas aeruginosa* biofilms to antimicrobial agents-how *P. aeruginosa* can escape antibiotics. Front Microbiol.

[10] Kohanski MA, DePristo MA, Collins JJ (2010). Sublethal antibiotic treatment leads to multidrug resistance via radical-induced mutagenesis. Mol Cell.

[11] Dwyer DJ (2014). Antibiotics induce redox-related physiological alterations as part of their lethality. Proc Natl Acad Sci U S A.

[12] Malhotra S, Hayes D, Wozniak DJ (2019). Cystic fibrosis and *Pseudomonas aeruginosa*: The host-microbe interface. Clin Microbiol Rev.

[13] da Cruz Nizer WS (2021). Oxidative stress response in *Pseudomonas aeruginosa*. Pathogens.

[14] Mandsberg LF (2011). Development of antibiotic resistance and up-regulation of the antimutator gene pfpI in mutator *Pseudomonas aeruginosa* due to inactivation of two DNA oxidative repair genes (mutY, mutM). FEMS Microbiol Lett.

[15] Kivisaar M (2010). Mechanisms of stationary-phase mutagenesis in bacteria: mutational processes in pseudomonads. FEMS Microbiol Lett.

[16] Bergkessel M, Basta DW, Newman DK (2016). The physiology of growth arrest: uniting molecular and environmental microbiology. Nat Rev Microbiol.

[17] Vallet-Gely I, Boccard F (2013). Chromosomal organization and segregation in *Pseudomonas aeruginosa*. PLoS Genet.

[18] Suzuki T (2006). DNA damage and mutation caused by vital biomolecules, water, nitric oxide, and hypochlorous acid. Genes Environ..

[19] Bassetti M, Vena A, Croxatto A, Righi E, Guery B (2018). How to manage *Pseudomonas aeruginosa* infections. Drugs Context.

[20] Drlica K (1999). Mechanism of fluoroquinolone action. Curr Opin Microbiol.

[21] Monti MR, Morero NR, Miguel V, Argarana CE (2013). nfxB as a novel target for analysis of mutation spectra in *Pseudomonas aeruginosa*. PLoS One.

[22] Morero NR, Argarana CE (2009). *Pseudomonas aeruginosa* deficient in 8-oxodeoxyguanine repair system shows a high frequency of resistance to ciprofloxacin. FEMS Microbiol Lett.

[23] Morero NR, Monti MR, Argarana CE (2011). Effect of ciprofloxacin concentration on the frequency and nature of resistant mutants selected from *Pseudomonas aeruginosa* mutS and mutT hypermutators. Antimicrob Agents Chemother.

[24] Zaborskyte G, Andersen JB, Kragh KN, Ciofu O (2017). Real-time monitoring of nfxB mutant occurrence and dynamics in *Pseudomonas aeruginosa* biofilm exposed to subinhibitory concentrations of ciprofloxacin. Antimicrob Agents Chemother.

[25] Fujita C, Nishimura A, Iwamoto R, Ikehara K (2002). Guanosine 5'-diphosphate 3'-diphosphate (ppGpp) synthetic activities on *Escherichia coli* SpoT domains. Biosci Biotechnol Biochem.

[26] Gaca AO, Colomer-Winter C, Lemos JA (2015). Many means to a common end: the intricacies of (p)ppGpp metabolism and its control of bacterial homeostasis. J Bacteriol.

[27] Viducic D (2006). Functional analysis of spoT, relA and dksA genes on quinolone tolerance in *Pseudomonas aeruginosa* under nongrowing condition. Microbiol Immunol.

[28] Irving SE, Choudhury NR, Corrigan RM (2021). The stringent response and physiological roles of (pp)pGpp in bacteria. Nat Rev Microbiol.

[29] Turner KH, Wessel AK, Palmer GC, Murray JL, Whiteley M (2015). Essential genome of *Pseudomonas aeruginosa* in cystic fibrosis sputum. Proc Natl Acad Sci USA.

[30] Lee SA (2015). General and condition-specific essential functions of *Pseudomonas aeruginosa*. Proc Natl Acad Sci USA.

[31] Poulsen BE (2019). Defining the core essential genome of *Pseudomonas aeruginosa*. Proc Natl Acad Sci USA.

[32] Jovanovic M, Lilic M, Janjusevic R, Jovanovic G, Savic DJ (1999). tRNA synthetase mutants of *Escherichia coli* K-12 are resistant to the gyrase inhibitor novobiocin. J Bacteriol.

[33] Garoff L, Huseby DL, Praski Alzrigat L, Hughes D (2018). Effect of aminoacyl-tRNA synthetase mutations on susceptibility to ciprofloxacin in *Escherichia coli*. J Antimicrob Chemother.

[34] Kim HY (1998). Alginate, inorganic polyphosphate, GTP and ppGpp synthesis co-regulated in *Pseudomonas aeruginosa*: Implications for stationary phase survival and synthesis of RNA/DNA precursors. Mol Microbiol.

[35] Bernardo LM, Johansson LU, Solera D, Skarfstad E, Shingler V (2006). The guanosine tetraphosphate (ppGpp) alarmone, DksA and promoter affinity for RNA polymerase in regulation of sigma-dependent transcription. Mol Microbiol.

[36] Viducic D, Murakami K, Amoh T, Ono T, Miyake Y (2016). RpoN modulates carbapenem tolerance in *Pseudomonas aeruginosa* through pseudomonas quinolone signal and PqsE. Antimicrob Agents Chemother.

[37] Pletzer D, Mansour SC, Hancock REW (2018). Synergy between conventional antibiotics and anti-biofilm peptides in a murine, sub-cutaneous abscess model caused by recalcitrant ESKAPE pathogens. PLoS Pathog.

[38] Viducic D (2007). rpoN gene of *Pseudomonas aeruginosa* alters its susceptibility to quinolones and carbapenems. Antimicrob Agents Chemother.

[39] Le S (2014). Chromosomal DNA deletion confers phage resistance to *Pseudomonas aeruginosa*. Sci Rep.

[40] Shen M (2018). *Pseudomonas aeruginosa* MutL promotes large chromosomal deletions through non-homologous end joining to prevent bacteriophage predation. Nucleic Acids Res.

[41] Dettman JR, Sztepanacz JL, Kassen R (2016). The properties of spontaneous mutations in the opportunistic pathogen *Pseudomonas aeruginosa*. BMC Genomics.

[42] Fedeles BI (2015). Intrinsic mutagenic properties of 5-chlorocytosine: A mechanistic connection between chronic inflammation and cancer. Proc Natl Acad Sci USA.

[43] Cirz RT (2005). Inhibition of mutation and combating the evolution of antibiotic resistance. PLoS Biol.

[44] Didier JP (2011). Impact of ciprofloxacin exposure on Staphylococcus aureus genomic alterations linked with emergence of rifampin resistance. Antimicrob Agents Chemother.

[45] Song LY (2016). Mutational consequences of ciprofloxacin in *Escherichia coli*. Antimicrob Agents Chemother.

[46] Jacobs MA (2003). Comprehensive transposon mutant library of *Pseudomonas aeruginosa*. Proc Natl Acad Sci USA.

[47] Klockgether J (2010). Genome diversity of *Pseudomonas aeruginosa* PAO1 laboratory strains. J Bacteriol.

[48] Sidorenko J, Jatsenko T, Kivisaar M (2017). Ongoing evolution of *Pseudomonas aeruginosa* PAO1 sublines complicates studies of DNA damage repair and tolerance. Mutat Res.

[49] Varadarajan AR (2020). An integrated model system to gain mechanistic insights into biofilm-associated antimicrobial resistance in *Pseudomonas aeruginosa* MPAO1. NPJ Biofilms Microbiomes.

[50] Su HC (2010). The development of ciprofloxacin resistance in *Pseudomonas aeruginosa* involves multiple response stages and multiple proteins. Antimicrob Agents Chemother.

[51] Cohen NR, Lobritz MA, Collins JJ (2013). Microbial persistence and the road to drug resistance. Cell Host Microbe.

[52] Luidalepp H, Joers A, Kaldalu N, Tenson T (2011). Age of inoculum strongly influences persister frequency and can mask effects of mutations implicated in altered persistence. J Bacteriol.

[53] Windels EM (2019). Bacterial persistence promotes the evolution of antibiotic resistance by increasing survival and mutation rates. ISME J.

[54] Balaban NQ (2019). Definitions and guidelines for research on antibiotic persistence. Nat Rev Microbiol.

[55] Irving SE, Corrigan RM (2018). Triggering the stringent response: signals responsible for activating (p)ppGpp synthesis in bacteria. Microbiology (Reading).

[56] Khare A, Tavazoie S (2020). Extreme antibiotic persistence via heterogeneity-generating mutations targeting translation. mSystems.

[57] Viducic D, Murakami K, Amoh T, Ono T, Miyake Y (2017). RpoN promotes *Pseudomonas aeruginosa* survival in the presence of tobramycin. Front Microbiol.

[58] Levinson G, Gutman GA (1987). Slipped-strand mispairing: a major mechanism for DNA sequence evolution. Mol Biol Evol.

[59] Mercolino J, Lo Sciuto A, Spinnato MC, Rampioni G, Imperi F (2022). RecA and specialized error-prone DNA polymerases are not required for mutagenesis and antibiotic resistance induced by fluoroquinolones in *Pseudomonas aeruginosa*. Antibiotics Basel.

[60] Hobbs JK, Boraston AB (2019). (p)ppGpp and the stringent response: An emerging threat to antibiotic therapy. ACS Infect Dis.

[61] Khakimova M, Ahlgren HG, Harrison JJ, English AM, Nguyen D (2013). The stringent response controls catalases in *Pseudomonas aeruginosa* and is required for hydrogen peroxide and antibiotic tolerance. J Bacteriol.

[62] Mwangi MM (2013). Whole-genome sequencing reveals a link between beta-lactam resistance and synthetases of the alarmone (p)ppGpp in Staphylococcus aureus. Microb Drug Resist.

[63] Doumith M, Mushtaq S, Livermore DM, Woodford N (2016). New insights into the regulatory pathways associated with the activation of the stringent response in bacterial resistance to the PBP2-targeted antibiotics, mecillinam and OP0595/RG6080. J Antimicrob Chemother.

[64] Strugeon E, Tilloy V, Ploy MC, Da Re S (2016). The stringent response promotes antibiotic resistance dissemination by regulating integron integrase expression in biofilms. mBio.

[65] Bryson D, Hettle AG, Boraston AB, Hobbs JK (2020). Clinical mutations that partially activate the stringent response confer multidrug tolerance in Staphylococcus aureus. Antimicrob Agents Chemother.

[66] Levin-Reisman I (2017). Antibiotic tolerance facilitates the evolution of resistance. Science.

[67] Santi I, Manfredi P, Maffei E, Egli A, Jenal U (2021). Evolution of antibiotic tolerance shapes resistance development in chronic *Pseudomonas aeruginosa* infections. mBio.

[68] Martins D (2018). Superoxide dismutase activity confers (p)ppGpp-mediated antibiotic tolerance to stationary-phase *Pseudomonas aeruginosa*. Proc Natl Acad Sci USA.

[69] Meylan S, Andrews IW, Collins JJ (2018). Targeting antibiotic tolerance, pathogen by pathogen. Cell.

[70] Pacios O (2020). (p)ppGpp and its role in bacterial persistence: New challenges. Antimicrob Agents Chemother.

[71] Nguyen D (2011). Active starvation responses mediate antibiotic tolerance in biofilms and nutrient-limited bacteria. Science.

[72] Marvig RL, Sommer LM, Molin S, Johansen HK (2015). Convergent evolution and adaptation of *Pseudomonas aeruginosa* within patients with cystic fibrosis. Nat Genet.

[73] Lopez-Causape C (2017). Evolution of the *Pseudomonas aeruginosa* mutational resistome in an international Cystic Fibrosis clone. Sci Rep.

[74] Das B, Bhadra RK (2020). (p)ppGpp metabolism and antimicrobial resistance in bacterial pathogens. Front Microbiol.

[75] Van den Bergh B (2016). Frequency of antibiotic application drives rapid evolutionary adaptation of *Escherichia coli* persistence. Nat Microbiol.

[76] Held K, Ramage E, Jacobs M, Gallagher L, Manoil C (2012). Sequence-verified two-allele transposon mutant library for *Pseudomonas aeruginosa* PAO1. J Bacteriol.

[77] Hmelo LR (2015). Precision-engineering the *Pseudomonas aeruginosa* genome with two-step allelic exchange. Nat Protoc.

[78] Bryksin A, Matsumura I (2013). Overlap extension PCR cloning. Methods Mol Biol.

[79] FastQC: A quality control tool for high throughput sequence data https://www.bioinformatics.babraham.ac.uk/projects/fastqc/, 2010.

[80] H., L. Aligning sequence reads, clone sequences and assembly contigs with BWA-MEM. *arXiv*, 1303.3997 (2013).

[81] Li H (2009). The sequence alignment/map format and SAMtools. Bioinformatics.

[82] Li H (2011). A statistical framework for SNP calling, mutation discovery, association mapping and population genetical parameter estimation from sequencing data. Bioinformatics.

[83] Danecek P (2021). Twelve years of SAMtools and BCFtools. Gigascience.

[84] McLaren W (2016). The ensembl variant effect predictor. Genome Biol.

[85] Rosche WA, Foster PL (2000). Determining mutation rates in bacterial populations. Methods.

[86] Lea DE, Coulson CA (1949). The distribution of the numbers of mutants in bacterial populations. J Genet.

[87] Eucast D. Determination of minimum inhibitory concentrations (MICs) of antibacterial agents by agar dilution. *Clin. Microbiol. Infect.* **6** (2000). 10.1046/j.1469-0691.2000.00142.x 11168187

[88] Hazan R, Maura D, Que YA, Rahme LG (2014). Assessing *Pseudomonas aeruginosa* Persister/antibiotic tolerant cells. Methods Mol Biol.

[89] Dean CR, Goldberg JB (2002). *Pseudomonas aeruginosa* galU is required for a complete lipopolysaccharide core and repairs a secondary mutation in a PA103 (serogroup O11) wbpM mutant. FEMS Microbiol Lett.

