# Supplementary material for: A role for the stringent response in ciprofloxacin resistance in Pseudomonas aeruginosa
Source: Sci Rep. 2024 Apr 13;14:8598. doi: 10.1038/s41598-024-59188-z (PMC11016087; doi:10.1038/s41598-024-59188-z)
Supplement: Supplementary file 4 — Supplementary Information 4. [file 41598_2024_59188_MOESM4_ESM.pdf]

A role for the Stringent Response in ciprofloxacin resistance in *Pseudomonas aeruginosa*

Libertad García-Villada[1]; Natalya P. Degtyareva[1]; Ashley M. Brooks[2]; Joanna B. Goldberg[3], Paul W. Doetsch[1]*

[1]Genomic Integrity & Structural Biology Laboratory, NIEHS, Durham, NC, USA.

[2]Integrative Bioinformatics, Biostatistics and Computational Biology Branch, NIEHS, Durham, NC, USA.

[3]Department of Pediatrics, Emory University School of Medicine, Atlanta, GA, USA.

ID: LG-V, 0000-0001-9796-9341; NPD, 0000-0002-7141-6308; AMB, 0000-0003-2242-5942; JBG, 0000-0002-5285-5188; PWD, 0000-0003-3195-3413

*Corresponding author

E-mail: paul.doetsch@nih.gov

Table S1. MIC values (μg/ml) for WT and ciproR mutant strains.

Strain	Cipro / Cipro comp.[a,b]	Tetracycline	Rifampicin	Gentamicin	Imipenem
**MPAO1**	1 / 1	25	30	1.5	6
**PAO1**	1 / 1	25	30	1.5	6
spoT1	4 / 1	20	30	0.5	4
spoT2	4 / 3	20	30	0.5	4
spoT3	4 / 1	20	30	0.5	4
spoT4	4 / 2	20	30	0.5	4
alaS	4 / 10	20	25	0.5	5
glyS	8 / nd	30	30	1.5	4
ileS	5 / nd	25	30	0.5	6
rpoN	6 / nd	30	40	1.0	6
**T83I**	15	25	35	1.5	6
**D87N**	10	25	35	1.5	6

[a]comp. means complemented.

[b]nd means not determined.

Table S2. List of primers used in this study.

PRIMER	SEQUENCE
spoT_PCR-F	TCGCCTAAATCGGCTCTTCC
spoT_PCR-R	GATGGCGTCCATTTCCACCT
spoT_seq1	AAAGTGGCCTGGGAAAACGA
spoT_seq2	GATATCCGCGTGATCCTGGT
spoT_seq3	GTACCCGCGAGATGGAAGAG
spoT_seq4	GACCTGCTGGAAGAGATCGG
alaS-PCR-F	CGTCGAACTGATCGGCAAGG
alaS-PCR-R	CACCTGGTTGCCGGTATAGG
alaS_seq1	CGAAAAGGTCGGCAATAGGC
alaS_seq2	CCCTATGCGTCCGACAACTT
alaS_seq3	ACCTGACCAATGACATCGCC
alaS_seq4	GAGGTCGAGACCGAGGAAAC
alaS_seq5	AAATCAAGGGCGCGAAGGTA
glyS_PCR-F	TCACCTATGGCGACGTGTTC
glyS_PCR-R	GAAAGCGTGATGACCGACGA
glyS_seq1	CCAGAACGAGGTGGAGCAAT
glyS_seq2	GCTGAAGGAGGCCGAATGAT
glyS_seq3	AACGAACTGCCGATTCCCAA
glyS_seq4	TGGTCGGTATCTTCGGCATC
glyS_seq5	TATTCTCGCCAAGTCCGAGG
rpoN_PCR-F	CGAAGGGTATCGGCATCCTC
rpoN_PCR-R	TCGAGTACGCGTTTCTTGCT
rpoN_seq1	CGAGACCCTGGACATCTGTG
rpoN_seq2	ATTTCGACAACAGCGACCCT
rpoN_seq3	GCGCATGAAGCTCAAGGAAG
rpoN_seq4	AAGTCACGTCAGTACCGCTG
ileS_PCR-F	GGTCCTGCATGGACAGAAAC
ileS_PCR-R	GCGGCTTCTCCGGATTTCTT
ileS_seq1	CGTCTACCTGGTCAGTACGC
ileS_seq2	TCATTGTCCGCTCCAAGACC
ileS_seq3	CTGAACGTGCATCCGGAGAT
ileS_seq4	CACAAGACGCCGCTGATCTA
ileS_seq5	GGTGGCTTCGACCGATTACT
ileS_seq6	GTAATGCTCGCCACCTGGTA
gyrA_PCR-F	CGTGGGCTGTGGTAAGCT
gyrA_PCR-R	ATGTACTGCATCAGCTCATCG
gyrA_seq-F	AGTTGGCGCTGCTCG
gyrA_seq-F	GCCCTGGCCGTCTACC
alaS_VECTOR-F SacI	CTACGAGCTCGCGAAAAGGTCGGCAATA
alaS_VECTOR-R XbaI	GTAGTCTAGATCAGAGCCCTTGCTCGA
spoT_VECTOR-F SacI	CTACGAGCTCATCGGCTCTTCCATAAAGAT
spoT_VECTOR-R XbaI	GTAGTCTAGATCAGCTACGCAGGCGGGTGA

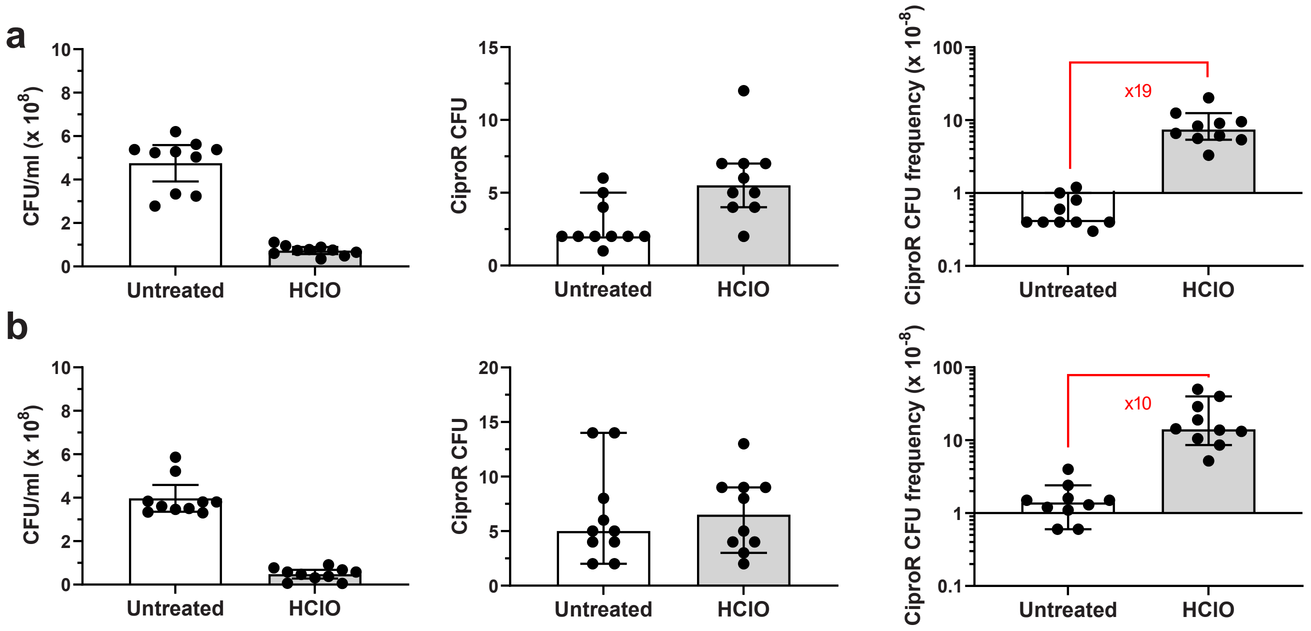

Figure S1. HClO exposure increases the frequency of ciproR mutants in *P. aeruginosa* stationary-phase cultures. Graphs in **a** and **b** summarize the results from two independent experiments. From left to right, the graphs represent (i) CFU/ml (mean +/- 95% CI) of untreated and HClO-treated; (ii) ciproR CFU (median +/- 95% CI) observed for untreated and HClO-treated cultures when 2 ml of each experimental culture was plated on the selective plates; and (iii) frequency (median +/- 95% CI) of ciproR CFU of untreated and HClO-treated cultures (n= 10). In both experiments, exposure to HClO determined a decrease in survival of about 7.5-fold; untreated and HClO-treated cultures had roughly the same numbers of ciproR CFU; and HClO-treated cultures showed an increase in the frequency of ciproR CFU.

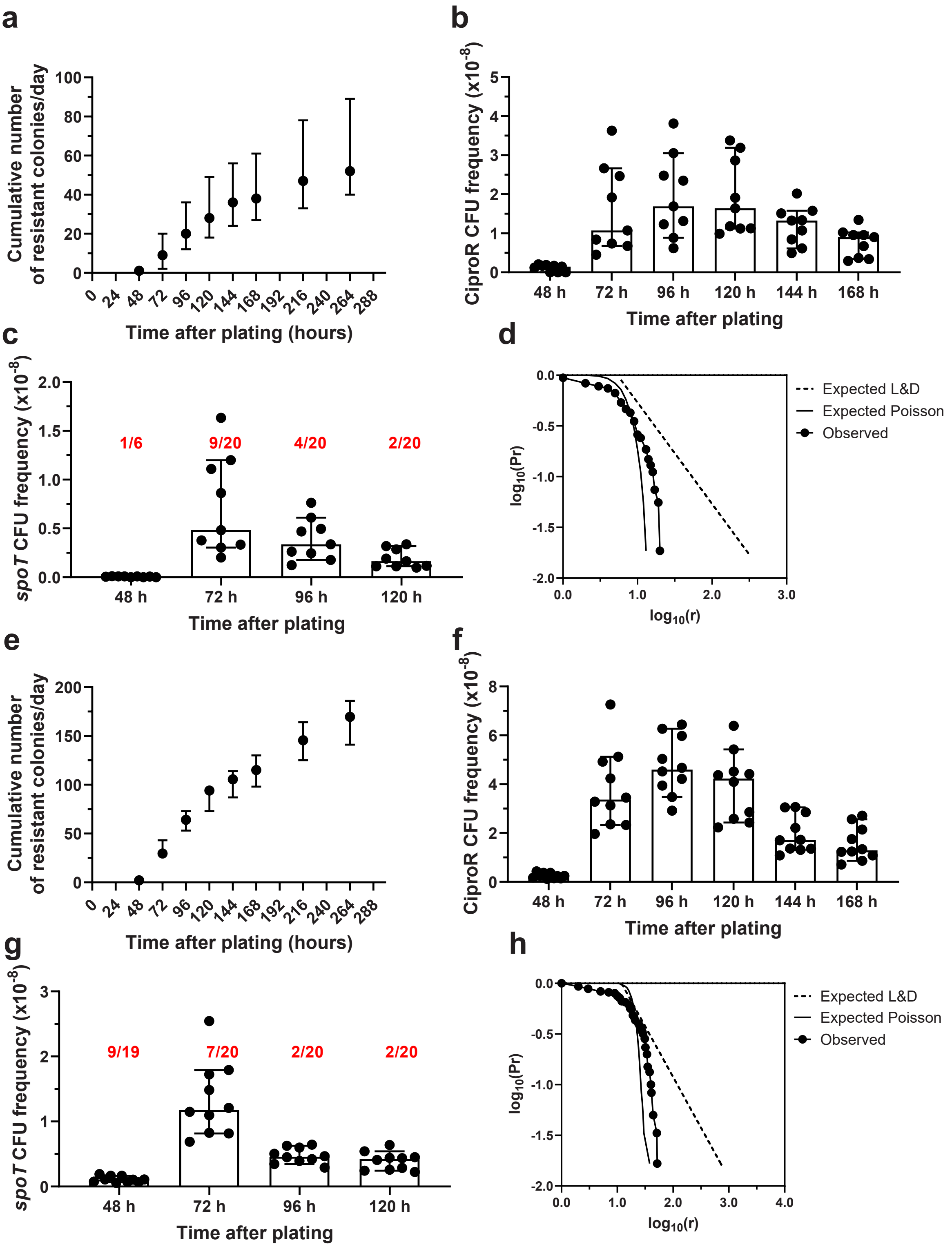

FigureS2. Evidence for induction of ciproR mutants by cipro. *P. aeruginosa* WT strain MPAO1 cultures were grown in minimal media and then plated on selective plates containing 2 μg/ml cipro. **a**, **b**, **c**, and **d**, represent the results for one experiment, and **e**, **f**, **g**, and **h**, represent the results for another. All these results are similar to those lengthy explained in the main text of the paper, Fig. 1, for a representative experiment.

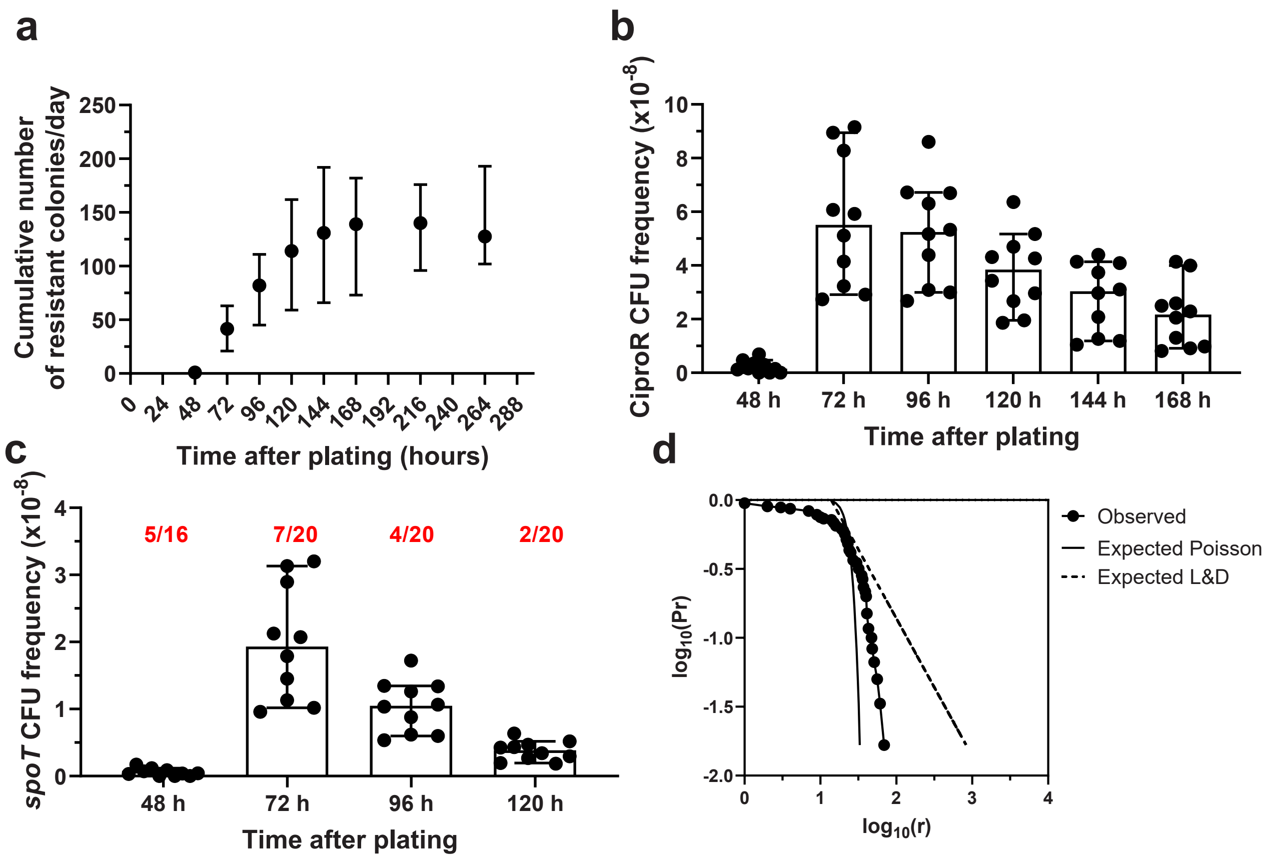

Figure S3. Evidence for induction of ciproR mutants by cipro in the *P. aeruginosa* WT strain PAO1. All these results are similar to those described in detail in the main text of the paper, Fig. 1

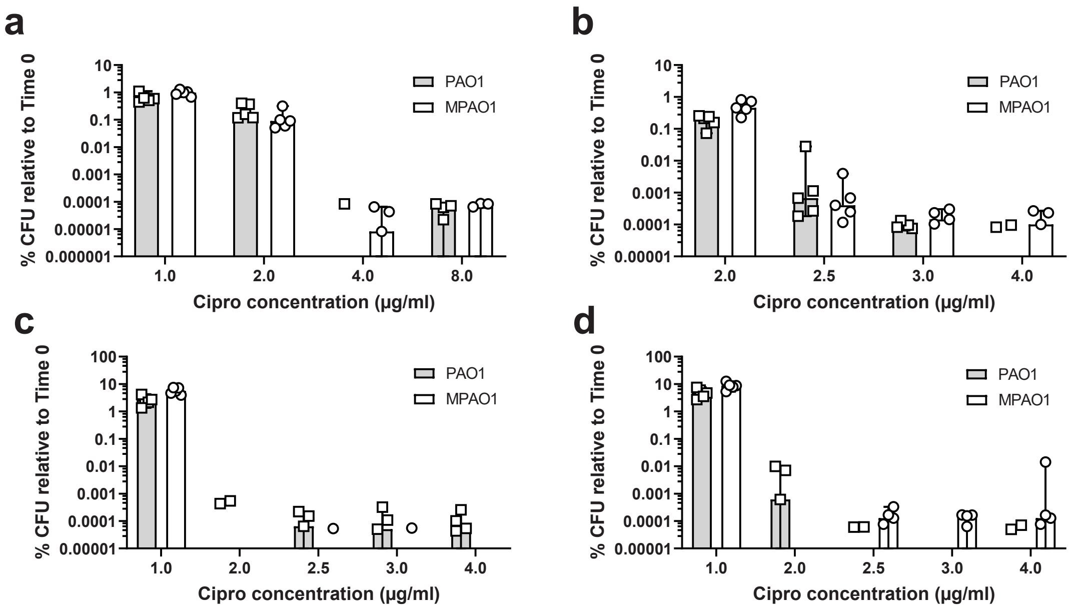

Figure S4. Minimal bactericidal concentration of cipro for WT cultures is in the range of 2 to 2.5 µg/ml and varies between experiments. We attempted to determine the minimum bactericidal concentration of cipro under our experimental conditions and, in particular, the effect of the selective concentration 2 µg/ml cipro**a**, **b**, **c**, and **d**, represent results from four different experiments. Bars and error bars represent median and 95% CI of n= 10.

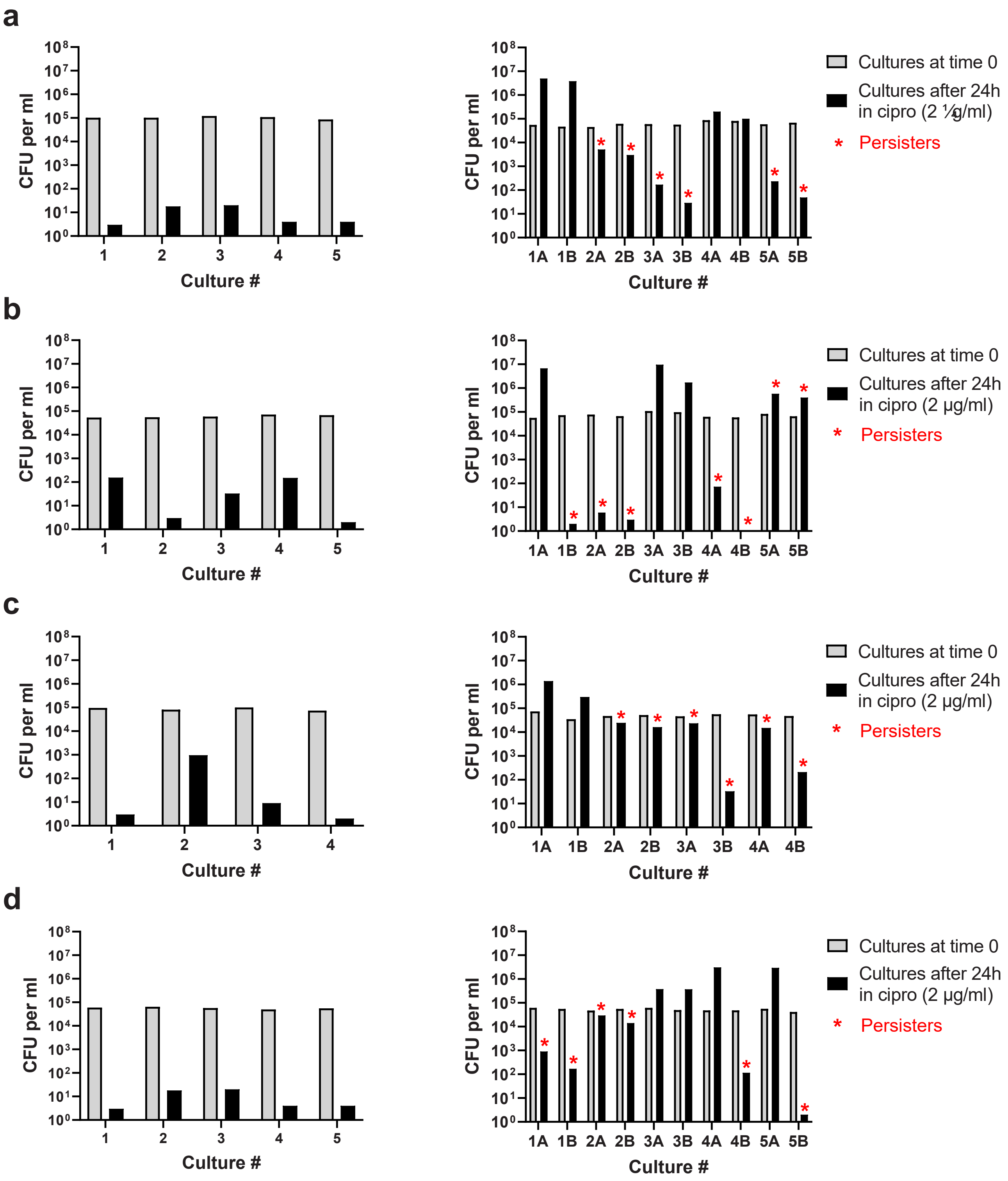

Strain	Experiment	Cipro MIC (µg/ml) 0	1.0 a)	1.5 a)	2.0 a)	4.0	10.0
**MPAO1**	**A**		6		4		
	**B**		7	1	2		
**PAO1**	**C**		6		2		
	**D**		6	3	1		

a) number of ciproR clones with the corresponding MIC

Figure S5. Persisters and genuine mutants comprise the population of ciproR isolates. MPAO1 and PAO1 isolates were incubated ON with 2 μg/ml of cipro and the number of survivors determined. A random sample of the survivors were then exposed again to 2 μg/ml of cipro, and the number of survivors and their cipro MIC determined. Graphs represent the number of cells/ml in the cultures at time 0, and at the end of each incubation. Bars and error bars represent median and range of n= 5 isolates. Table represent the MIC values for 10 independent survivors isolated per strain. **a**, **b**, **c**, and **d** summarize the results from four independent experiments (**a** and **b** conducted with WT strain MPAO1, and **c** and **d** conducted with WT strain PAO1). Graphs on the left represent the results of the first round of cipro exposure, and the graphs on the right represent the second round. The second round was accomplished with two independent isolates (A and B) from each experimental culture of the first round. Most isolates that survived the second round of exposure have the same cipro MIC as the corresponding parental, WT strain. Accordingly, we hypothesized that these survivors are persisters. See text for additional details.

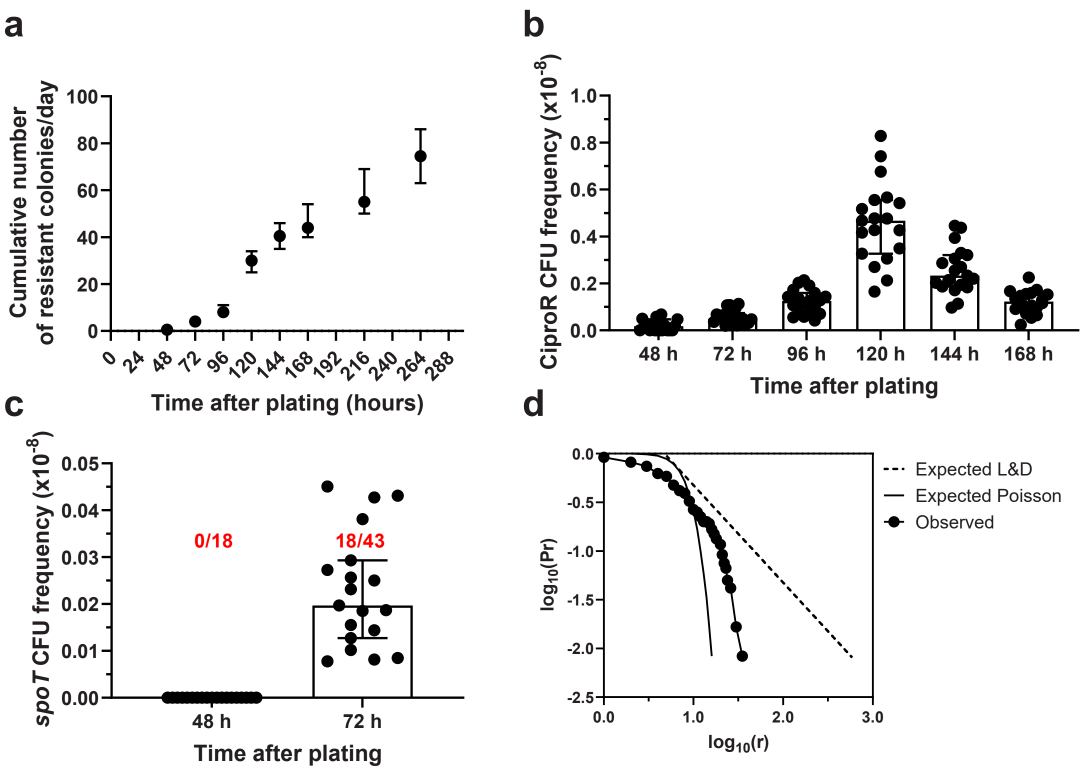

Figure S6. Standard selection delays but does not prevent the appearance of SR mutants. *P. aeruginosa* WT strain MPAO1 cultures were grown in LB and then plated on selective plates containing 2 µg/ml cipro. **a**, **b**, **c**, and **d** represent the results for one experiment. All of these results are similar to those described in detail in the main text of the paper, Fig. 3.

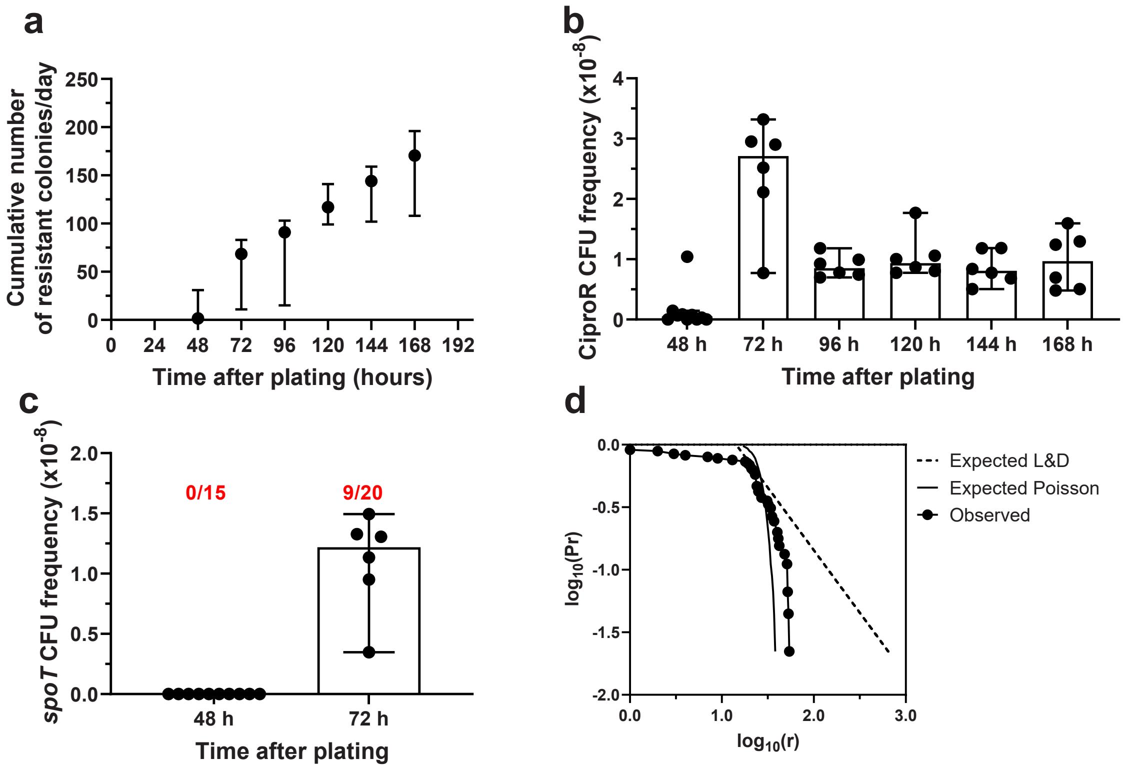

Figure S7. Standard selection delays but does not prevent the appearance of SR mutants for the *P. aeruginosa* WT strain PaO1. All of these results are similar to those described in detail in the main text of the paper, Fig 3.

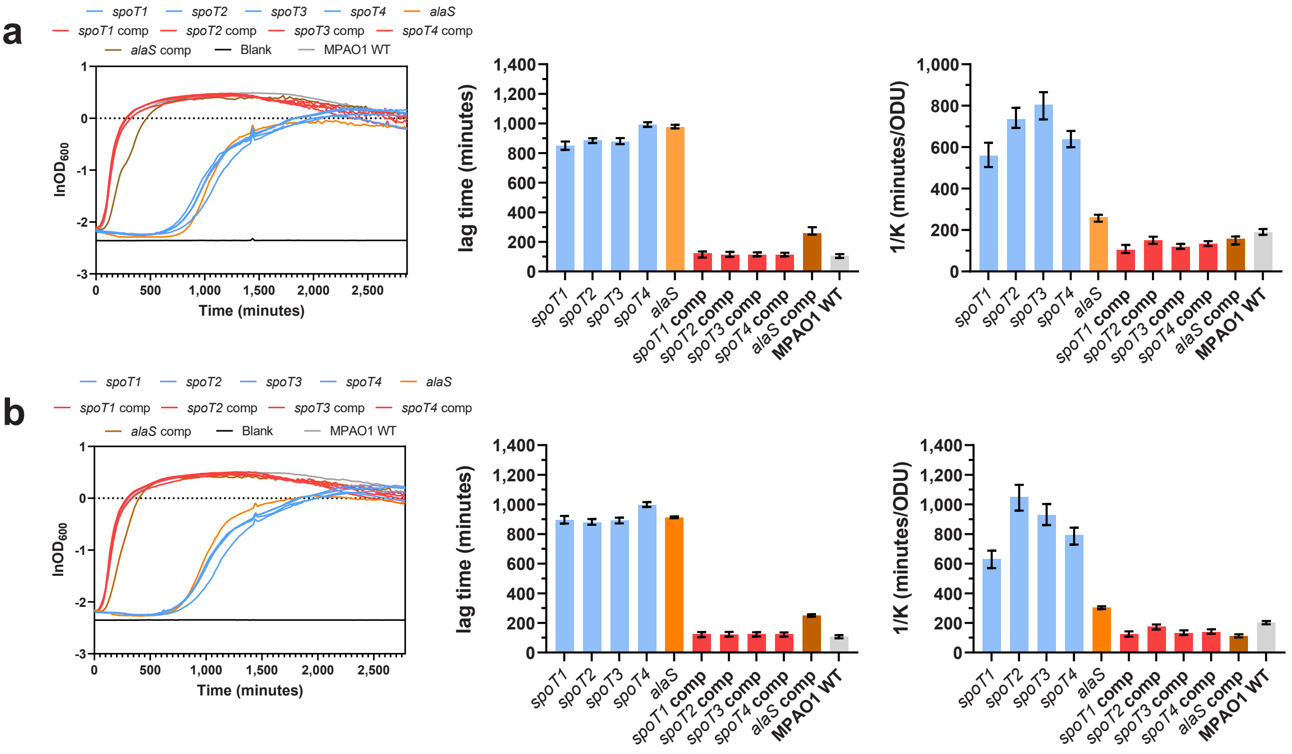

Figure S8. Genetic complementation restores growth impairment in SR mutants. Graphs in **a** and **b** summarize the results from two independent experiments. From left to right, the graphs represent (i) growth curves; (ii) lag time; and (iii) growth rate (reverse) of different SR mutants (complemented and not complemented) and the corresponding WT strain (n= 8 technical replicates per strain per experiment).
